# Effects of repeated sterilization cycles on the surface alterations of ProTaper Next, TF Adaptive, HyFlex CM, and 2Shape instruments

**DOI:** 10.34172/joddd.2021.013

**Published:** 2021-05-05

**Authors:** Olcay Özdemir, Sibel Koçak, Mustafa Murat Koçak, Baran Can Sağlam

**Affiliations:** ^1^Department of Paediatric Dentistry, Faculty of Dentistry, Zonguldak Bülent Ecevit University, Turkey; ^2^Department of endodontic Dentistry, Faculty of Dentistry, Zonguldak Bülent Ecevit University, Turkey

**Keywords:** 2Shape, Atomic force microscope, HyFlex CM, ProTaper Next, Sterilization, TF-Adaptive

## Abstract

**Background.** This study aimed to evaluate the effects of repeated sterilization cycles on the surface alterations of various nickel-titanium instruments, including ProTaper Next (PTN), TF Adaptive (TFA), HyFlex CM (HCM), and 2Shape (2S).

**Methods.** Twenty-four new NiTi files of four different alloys were selected. The instruments in each group were divided into two equal groups, as follows: control and sterilization. The first group was chosen as the control without applying any sterilization procedure, while in group 2, five cycles of sterilization procedures were applied. The surface topographies were evaluated using scanning electron microscope (SEM) and atomic force microscope (AFM). The root mean square (RMS) and maximum height (MH) values and three-dimensional images were recorded. The data were analyzed with the Shapiro-Wilk test, one-way ANOVA, and post hoc Tukey tests. The statistical significance level was set at *P* ≤ 0.05.

**Results.** Before the experiment, HCM demonstrated the highest RMS value, and 2S showed the lowest. After the procedures, the RMS and MH values deteriorated on the surface of PTX, TFA, and 2S (*P* < 0.001). The HCM was not affected by sterilization processes (*P* > 0.05).

**Conclusion.** The initial irregularity on the surface did not affect the rate of alteration. The HCM files demonstrated superior surface properties after several cycles of sterilization. The PTN, TFA, and 2S presented similar surface responses after five cycles of autoclave sterilization.

## Introduction

The infection control protocol is the most critical factor that makes instruments safe for re-use. Recently, there is no consensus on the number of uses for rotary NiTi instruments.^1^ Some manufacturers recommended the single use of NiTi files; however, files are frequently re-used after autoclave sterilization.^2^ The chemical and physical reactions that occur during disinfection and sterilization might cause corrosion and deterioration on the instruments’ surface, leading to surface alterations, early fracture, and decreased cutting efficacy.^3,4^ The operational speed, motion principle, metallurgical properties, and surface characteristics are substantial factors that can affect instrument fatigue.^5^

Different thermally treated NiTi alloys, like CM-Wire, M-Wire, T-Wire, and R-phase, have been introduced to optimize the transformation behavior of NiTi alloy microstructure, which affects the mechanical structure.^6-8^ ProTaper Next (PTN: Dentsply Tulsa Dental Specialties, Tulsa, OK) is a multi-file preparation system manufactured by M-wire alloy that underwent a specific thermomechanical process.^9^ TF Adaptive (TFA: SybronEndo, Orange, CA, USA) is also a multi-file system manufactured by a twisting method using a special heat treatment R-phase of NiTi alloy.^5^ HyFlex CM (HCM: Coltene/Whaledent, Inc, Cuyahoga Falls, OH, USA) is a multi-file preparation system and manufactured by CM-Wire, which is a shape memory NiTi termed ‘Controlled Memory.’ This file system is produced by a lower percentage of the nickel weight than most commercially available NiTi and recommended for multi-use after sterilization as claimed by the manufacturer.^10,11^ 2Shape (2S; Micro-Mega, Besancon, France) is a double-file system manufactured by T-Wire alloy, produced with proprietary heat treatment.^6^

Stereomicroscopic scanning electron microscope (SEM) and atomic force microscope (AFM) are used to evaluate the file surface structure. Nowadays, AFM has become popular in providing three-dimensional reconstruction and qualitative and quantitative information. To date, only one study has assessed the effect of autoclave sterilization on the surface deterioration of the HCM file system using AFM.^12^ However, no evaluation of the surface characteristic of PTN, TFA, and 2S files has been performed. This study aimed to evaluate and compare the effects of repeated sterilization cycles on the surface alterations of PTN, TFA, HCM, and 2S instruments made of different alloys with different properties and production methods.

## Methods

Twenty-four new NiTi instruments of four different alloys were selected. According to alloys, the instruments were divided into four equal groups, including six samples: PTN #25/0.06, TFA #25/0.06, HCM #25/0.06, and 2S #25/0.06 were used in the study. The instruments were examined with the naked eye and under a stereomicroscope (Olympus SZ61 Stereomicroscope: Olympus, Tokyo, Japan) under ×20 magnification for any existing defects. The instruments with any deformation were excluded. The groups were randomly divided into two subgroups. Group 1 was selected as the control without applying any disinfection or sterilization procedures, while in group 2, five cycles of sterilization procedures were applied. The sterilization was performed at 121°C and 15 psi for 20 minutes after ultrasonic cleaning (SMB-SSD-450 Steam Sterilizers, Sterilmed Medical, Ankara, Turkey). The instruments were allowed to cool and dry at room temperature after sterilization procedures.

The 5-mm tip of each file was separated. The specimens were mounted on a metal base with rapid-drying cyanoacrylate glue. Each sample was placed in the AFM (Multi-Mode 8, Veeco Ins., Santa Barbara, CA, USA) for topographic evaluation. The surface analysis was performed on 11 different regions from the 3-mm tip of the instrument. The digital AFM images (5×5 μm) of the samples were obtained using the non-contact mode operation under ambient conditions. The root mean square (RMS) and MH values of the surface topography were recorded for evaluating the alterations on surfaces caused by the sterilization. Initial energy dispersive x-ray spectrophotometry (EDX) analysis of files and SEM images were recorded.

Statistical analyses were performed with SPSS 23 (SPSS, IBM Corp., New York, USA). Data were presented as means and standard deviations (SD). First, the RMS and depth data were analyzed using the Shapiro-Wilk test to verify the assumption of normality. One-way ANOVA and post hoc Tukey tests were used to compare the systems. The statistical significance level was set at *P* ≤ 0.05.

## Results

**Figure 1:**
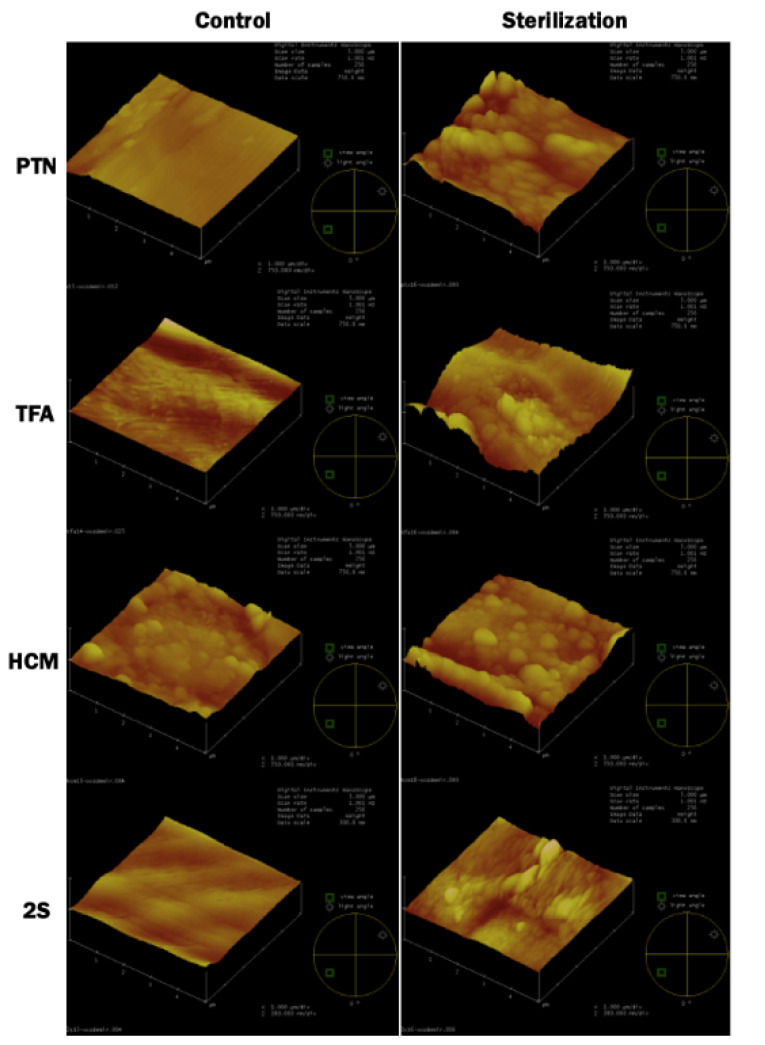
Representative AFM images of the tested files.

**Figure 2:**
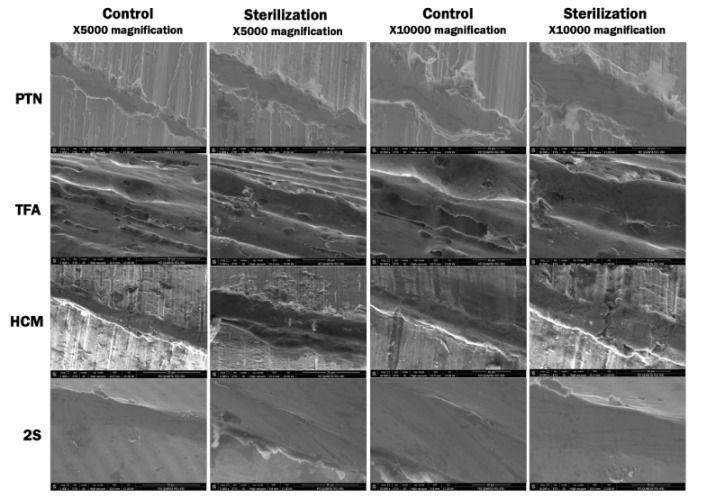
Representative SEM images of the tested files at various magnifications.

**Figure 3:**
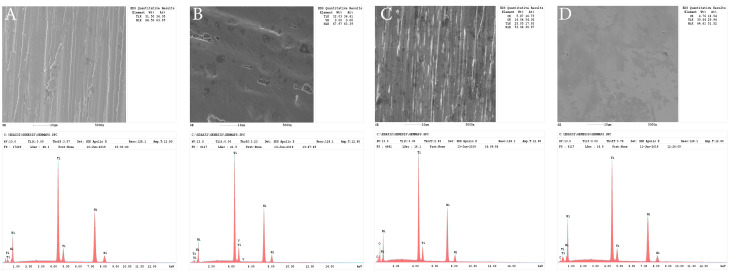
Representative EDX analysis of the tested files (a. PTN, b. TFA, c. HCM, and d. 2S).

The control group of HCM demonstrated the highest RMS value, and 2S exhibited the lowest. After sterilization procedures, the RMS and MH values showed deterioration on the surface of PTX, TFA, and 2S files, which was statistically significant (*P* < 0.001), but no difference was observed between PTX, TFA, and 2S (*P* > 0.05). The HCM instruments were not affected by sterilization processes compared to the control group (*P* > 0.05) (Table 1, Figure 1). The three-dimensional AFM, SEM, and EDX images of all groups are presented in Figures 1, 2, and 3.

**Table 1:** Means and standard deviations of MH and RMS; apical third of the active part in nm

	**MH**		**RMS**	
	**Control**	**Sterilization**	**Control**	**Sterilization**
**(M±SD)**	**(M±SD)**	**(M±SD)**	**(M±SD)**
PTN	140.97 ± 84.01	234.96 ± 120.7^*a^	32.06 ± 20.78	51.95 ± 25.75^*b^
TFA	103.63 ± 40.96	223.43 ± 123.31^*a^	23 ± 8.73	44.45 ± 23.55^*b^
HCM	215.3 ± 90.05	230.35 ± 79.75	43.46 ± 18.46	43.92 ± 20.04
2S	64.12 ± 27.56	123.99 ± 68.3^*a^	12.06 ± 6.89	26.25 ± 20.83^*b^
*P* value	*P* < 0.001^*^		*P* < 0.001^*^	

* Value that differs from the control in each group (*P* < 0.001) (M: Mean value, SD: Standard deviation) ^a,b^Significant between groups set at *P* ≤ 0.05. Means with the same letters indicate no difference, statistically significant between groups.

## Discussion

Instruments commonly used in dental practice to perform endodontic treatments are re-usable after sterilization by autoclaving.^3,13^ Although single-use of nickel-titanium files is safe and widely recommended during root canal preparation, it has been reported that NiTi files could be used up to three times in root canals.^4^ However, various studies reported high corrosion rates on file surfaces due to autoclaving at high temperatures.^14,15^

The surface evaluation of endodontic files can be evaluated by stereomicroscopy, SEM, and AFM.^16-18^ AFM provides sensitive and reliable data during file surface characterization.^2^ Therefore, AFM was used (in non-contact mode) to provide numerical data and display 3D images in the present study. The instruments can be analyzed both in non-contact or contact mode. However, in contact mode, the surface might be affected by lateral forces, causing unexpected variations in the samples’ height measurement.^19^

To the best of our knowledge, an increase in surface deterioration was associated with the breakage of NiTi files during clinical use, especially in curved canals.^2,20^ Laboratory studies have limitations to mimic clinical conditions. However, it is difficult to determine the exact reason for the surface topography changes in AFM studies, especially in evaluating the file surface.^21^ Therefore, in this study, only sterilization was applied to reflect the effect of autoclaving procedures on the files, independent of various impacts during the clinical use, including irrigation and instrumentation.

Previous studies have evaluated the effect of several sterilization cycles.^1,2,12,22^ However, a comprehensive systematic review concluded that statistically significant corrosion and micro-pitting occurred after five autoclave cycles.^3^ Therefore, five rounds of sterilization procedures were evaluated in this study.

Developments in NiTi file metallurgical technologies from the past to the present provided significant improvements to increase the elasticity, strength, and surface quality. The alloy’s mechanical behavior and properties vary based on its thermal/mechanical treatment during manufacturing and its chemical composition.^9,23^ According to Thompson,^24^ the NiTi alloy’s unique properties are associated with a solid-state phase transformation named the martensitic transformation. The temperature reduction might induce martensitic transformation, with no changes in the matrix chemical composition, but with a macroscopic difference in the shape of the material. The transformation occurs between the martensitic and austenite phases.^23^ Besides, there is an intermediate trigonal phase between austenite and martensitic phases, named the R-phase.^25^ In the present study, the effects of sterilization procedures on the surface properties of four different brands of NiTi files with varying properties of the alloy were evaluated at room temperature, including M-wire (both martensitic and R-phase), R-phase, CM-wire (composite of R-phase, martensitic, and austenite), and T-wire (high austenite).^23,26^ The transition between phases due to temperature also depends on the heat treatment process and the alloy’s chemical composition.^27^

Several studies have evaluated surface alterations due to sterilization and its effect on the mechanical behavior of HCM.^12,28^ However, there is no information about the surface deterioration of PTN, TFA, and 2S files to the best of our knowledge. The HCM files are manufactured from CM-wire alloy, providing a different surface property compared to other tested files, and these instruments also contain less Ni (52%) compared to conventional alloys. At room temperature, HCM might be a composite of all phases; however, recent studies claim that the final austenite-phase manufacturing temperature is generally higher than room temperature.^29,30^ The manufacturer claims that HCM files return to their original shape at 134°C even after deformity due to usage.^10^ This claim was supported by many studies, consistent with our findings, that autoclave sterilization does not irreversibly affect the mechanical properties of the HCM files.^12,28,31^ The transition to the austenite-phase resulting from high temperatures, such as sterilization, might prevent deformation on the file surface. It might be explained by the difference in Ni concentration of the body of the files. Rapisarda et al^15^ and Thierry et al^32^ reported that Ni concentration on the file surface reduced with exposure to heat sterilization, and titanium oxide increased, indicating that oxidation might occur on the surface. It might be claimed that the increase of the austenite phase or a lower ratio of Ni in the file structure might decrease surface deformation.

The analysis showed a significant increase in RMS and MH values on the surface of PTN, TFA, and 2S compared with the control groups after five autoclave sterilization cycles. The degree of increase in surface alteration was not different for these files after five sterilization cycles, which was statistically significant. Both PTN, TFA, and 2S files were manufactured by proprietary heat treatment. This might be the explanation for their metallurgic response to high temperatures. 2S file demonstrated lower surface deterioration before and after the sterilization process. Higher austenite composition during the T-Wire alloy manufacturing process might enable the 2S instruments to have a softer structure at the test temperature. It is known that the martensitic phase is readily deformable and can be induced by temperature or stress.^27^

The AFM evaluation of the files showed that the new and subjected samples had irregularities and structural defects on the surface, such as pits and grinding slots. These findings were also supported by past studies.^21,33^ The initial analysis of the HCM file demonstrated the most significant roughness in this study, where 2S was the lowest. However, when HCM was subjected to the sterilization procedure, no considerable surface alteration was recorded. 2S surface characteristic had the most insufficient roughness both at the initial and after the sterilization procedure. Therefore, it might be claimed that the initial irregularity has no effect on the rate of deformation by the experiment.

Similar to the present results, previous AFM studies concluded that autoclave sterilization generally increased NiTi file surface roughness.^1,2,12,22^ Besides, Khabadze et al^34^ reported that repeated sterilization cycles increased the internal deformation of the NiTi alloy structure. However, the clinical implications of the studies are essential. Hilfer et al^35^ evaluated the effects of autoclave sterilization on the cyclic fatigue resistance of M-wire (GT Series X files, Dentsply Tulsa Dental Specialties, Tulsa, OK) and R-phase (Twisted Files, SybronEndo, Orange, CA) files. After three autoclave cycles, the M-wire’s cyclic fatigue values ​​were not affected, but the R-phase (25/.06) decreased. The present results showed that five autoclave cycles affected M-wire (PTN) and R-phase (TFA) file surfaces. The difference between the results might be due to the number of sterilization cycles. Unlike Hilfer et al,^35^ Özyürek et al^36^ reported that PTN files exhibited a statistically significant increase in the cyclic fatigue resistance after autoclave sterilization. The authors attributed this increase to heat treatment during the manufacturing process. Kim et al^37^ also evaluated cyclic fatigue and torsional fracture resistance of various NiTi instruments before and after sterilization at room temperature. The study included NiTi instruments with a tip size of #25, consisting of ProTaper Universal (Dentsply Maillefer, Ballaigues, Switzerland), K3XF (SybronEndo, Orange, CA, USA), TFA, and HyFlex EDM (Coltène/Whaledent, Inc., Altstäten, Switzerland) files. It was concluded that the autoclave sterilization generally did not significantly influence the torsional fracture and cyclic fatigue resistance. However, the authors reported that the temperature of experiments, such as the room or body temperature, might have affected the results. Although the CM-Wire alloy presented consistent results in our evaluation compared with Kim et al,^37^ the TFA file was deformed in the present study, similar to previous studies.^35^ A systematic review and meta-analysis of the effects of sterilization on the torsional properties of NiTi instruments found that autoclave sterilization reduced the torsional movement and increased the risk of file fracture but with low significance.^38^

AFM evaluation revealed the preliminary results of the effects of heat sterilization on file surfaces. It is believed that for the clinical significance and the limitations of the experimental setup, further investigations are required to assess its correlation with clinical implications like the risk of fracture, cutting efficiency, and metallurgic response.

## Conclusion

It is crucial to evaluate the effects of multiple sterilization cycles on the rotary file systems to minimize mechanical complications during endodontic treatment. Within the limitations of the study, the initial irregularity on the surface did not affect deformation severity. The HCM files demonstrated superior surface properties after several cycles of heat sterilization. The PTN, TFA, and 2S exhibited similar surface responses after five cycles of autoclave sterilization.

## Authors’ Contributions

All authors contributed to the study concept and design, material preparation, and data collection and analysis. All the authors commented on previous versions of the manuscript. All the authors read and approved the final manuscript.

## Acknowledgments

None.

## Funding

The study was supported by the Scientific Research and Development Department of Zonguldak Bülent Ecevit University (Grant number: 2017-27194235-05).

## Competing Interests

The authors declare no conflict of interests.

## Ethics Approval

Not applicable.
